# Genetic defects in common variable immunodeficiency

**DOI:** 10.1111/j.1744-313X.2007.00681.x

**Published:** 2007-08-01

**Authors:** O Kopecký, Š Lukešová

**Affiliations:** Second Department of Internal Medicine, Charles University in Prague, Faculty of Medicine, University Hospital Hradec Králové, Czech Republic

## Abstract

Common variable immunodeficiency (CVID) is the most frequent clinically manifested primary immunodeficiency. According to clinical and laboratory findings, CVID is a heterogeneous group of diseases. Recently, the defects of molecules regulating activation and terminal differentiation of B lymphocytes have been described in some patients with CVID. In this study, we show the overview of deficiencies of inducible costimulator, transmembrane activator and calcium-modulator and cytophilin ligand interactor, CD19 molecules, their genetic basis, pathogenesis and clinical manifestations.

## Introduction

Common variable immunodeficiency (CVID) is a heterogeneous group of diseases characterized by hypogammaglobulinemia and repeated, predominantly, bacterial infections. Most of the patients have the normal number of peripheral T and B lymphocytes. B lymphocytes, however, show defects of differentiation and proliferation after antigen stimulation. The number of plasma and memory cells is reduced. As a result of this, the values of serum IgG and IgA are reduced and also irregularly the values of IgM (Di Renzo *et al*., 2004). The antibody response to protein and polysaccharide antigens is insufficient or completely missing in some cases. The attempts to classify CVID based on in vitro immunoglobulin (Ig) production and B lymphocyte subsets have confirmed marked heterogeneity of this antibody defects (Weiler & Bankers-Fulbright, 2005). Differentiation of precursor cells into mature B lymphocytes and then into plasma cells is a process regulated by products of a number of genes. Mutation of genes for inducible costimulator (ICOS), the first single gene defect, has been described in patients with CVID (Grimbacher *et al*., 2003). Subsequently, the mutations of genes encoding transmembrane activator and calcium-modulator and cytophilin ligand interactor (TACI) and CD19 have been found.

## ICOS deficiency

**Figure 1:**
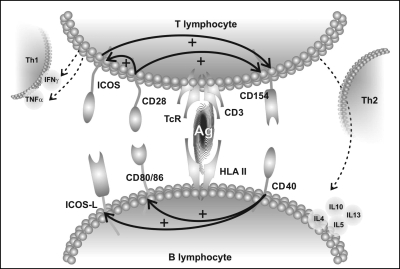
The cascade of co-stimulation signals between T and B lymphocytes. The expression of CD154 (CD40L) and ICOS is up-regulated through constitutively surface expressed CD28 molecule and its CD80/CD86 ligands. CD40-CD154 ligand amplifies the expression of CD80/CD86 and ICOS-L. By this another support of co-stimulation ligands between T and B lymphocytes occurs through ICOS-ICOS-L which up-regulates CD154 expression.

The ICOS molecule was described by Hutloff *et al*. (1999). It ranks in the family of costimulation molecules similarly as CD28 and CTLA-4 (CD152) molecules. It is encoded by three genes that are mapped to chromosome 2q33. A great number of single nucleotide variants that participate in the ICOS polymorphisms have been reported. The comparison of the frequency of single nucleotide polymorphisms between patients and healthy controls showed no significant differences (Haaning Andersen *et al*., 2003; Guzman *et al*., 2005; Ohm-Laursen *et al*., 2005). The ICOS and CD28 molecules share up to 20% amino acid homology and both are type I transmembrane receptors. ICOS occurs as a homodimer, with an extracellular (Ig) V like domain. Unlike the CD28 molecule, which is expressed constitutively, the ICOS molecule is expressed only on activated T lymphocytes. The ICOS expression is up-regulated within hours after TcR engagement. Its membrane expression persists on recently activated as well as on memory Th1 and Th2 CD4^+^ lymphocytes (van Berkel & Oosterwegel, 2006; Warnatz *et al*., 2006). The ICOS expression appears to be higher on Th2 cells compared to Th1 CD4^+^ T cells (Coyle *et al*., 2000). The Th2-mediated response is more dependent on the ICOS function than Th1 response (Löhning *et al*., 2003; Grunig *et al*., 2005). The density of ICOS membrane expression correlates with cytokine production. In case of ICOS gene mutation, especially the synthesis of cytokine associated with high-level ICOS expression (IL-10, IL-2, IL-4, IL-5, IL-13) is disturbed. Within the course of viral or bacterial infections in knock-out ICOS^−^/^−^ mice, large secondary germ centres fail to develop, and specific antibody response is missing. Both the reduced proliferation Th2 activity and the failure of T and B lymphocyte co-operation participate in the developed antibody defect (van Berkel & Oosterwegel, 2006; Warnatz *et al*., 2006) (Figure 1).

Triggering of TcR with appropriate costimulation leads to gene transcription in the nucleus. The intracellular tail of the ICOS molecule contains two tyrosine residues, one of which lies within the Y_Tyr180_MEM motive, which is associated with adapter molecule grow factor receptor-bound protein-2 (Grb-2). One amino acid replacement at the position in this motive includes explaining of the lack of capability to recruit Grb-2. This molecule plays a key role during the initiation of IL-2 synthesis (Harada *et al*., 2003). This finding explains why activating signals coming through defective ICOS molecules do not lead to synthesis of IL-2 (Itoh *et al*., 2002).

Activated T lymphocytes amplify the ICOS-L expression on B lymphocytes through IL-4, IL-5, IL-6, granulocyte macrophage colony-stimulating factor (GM-CSF), IFNγ and tumour necrosis factor (TNF)α cytokines (Bayry *et al*., 2005). ICOS-L expression on B lymphocytes is also up-regulated through CD40 and CD154 ligands or bacterial superantigens (Salzer *et al*., 2004; Janke *et al*., 2006). The ICOS: ICOS-L interaction is necessary for IL-10, IL-17 synthesis (Chtanova *et al*., 2004; Warnatz *et al*., 2006). The ICOS-L molecule is encoded by one gene, which is found on the long arm of chromosome 21 (mapped 21q22.3). It exists in two splice variants that differ in cytoplasmatic tail. The B7H2 variant is encoded by exon 1–7 and appears on both lymphoid and non-lymphoid cells. The hGL-50 variant is encoded by exon 1–6 and 8, and is found only on lymphoid cells (Ohm-Laursen *et al*., 2005; Warnatz *et al*., 2006). The ICOS-L is the receptor only for ICOS. Interaction of ICOS — ICOS-L molecules is important for differentiation of naive B lymphocytes into memory and plasma cells. ICOS-L ligand shares up to 20% homology with CD80 and CD86 molecules (receptors for CD28 and CTLA-4 molecules) (Salzer *et al*., 2004; van Berkel & Oosterwegel, 2006).

The homozygous deletion of ICOS causes an antibody deficiency syndrome in affected individuals. The clinical phenotype includes recurrent and serious bacterial infections, autoimmune phenomena, splenomegaly, lymphadenopathy and malignancy. Patients with CVID, based on ICOS defect, have mild B lymphopaenia and significantly reduced number of memory CD19^+^/CD27^+^ B lymphocytes (Warnatz *et al*., 2002; Piqueras *et al*., 2003). This laboratory finding is not, however, characteristic for ICOS mutations because up to 75% of patients with CVID have a reduced number of memory isotype-switched CD19^+^/CD27^+^/IgM^−^ B lymphocytes (Schejbel *et al*., 2005). However, patients with ICOS deficiency belong to CVID type I group (Freigburg classification) (Warnatz *et al*., 2002; Carsetti *et al*., 2005). ICOS deficiency, based on one point mutation, was the first example of an autosomal recessive disorder that occurs only in a small amount of patients with CVID (approximately 2%) (Warnatz *et al*., 2006).

## TACI deficiency

In order to ensure physiological antibody response after antigen stimulation, other activation signals mediated by both intracellular interactions and soluble factors are important (Kroczek *et al*., 2004). Class switch recombination (CSR) of Ig synthesis requires four signals. The first one is mediated by IL-3, IL-4, IL-5, IL-6, IL-13 cytokines, which participate in C_H_ genes transcription regulation. In case of a T-cell–dependent antibody response, the interaction of CD40L (CD154) molecule expressed on activated T lymphocytes with CD40 molecule on B lymphocytes is necessary (McHeyzer-Williams, 2003). Costimulatory ICOS — ICOS-L interaction constitutes the third necessary stimulus for sufficient antibody production. The fourth signal is mediated by other interactions of molecules, which belong to the superfamily of TNF molecules (B-cell–acting factor of the TNF family (BAFF), A proliferation-inducing ligand (APRIL)), and their receptors (B-cell maturation antigen (BCMA), BAFF receptor (BAFF-R), TACI).

The BAFF and APRIL are among these molecules (Castigli *et al*., 2005a). BAFF is present predominantly on monocytes/macrophages, dendric cells and T lymphocytes (Mackay & Ambrose, 2003). On the contrary, it is not expressed on B lymphocytes although this molecule has been detected on B-cell–derived chronic lymphatic leukaemia (Novak *et al*., 2002). The APRIL molecule is expressed on a large variety of cells, monocytes/macrophages, dendritic cells and activated T lymphocytes. BAFF and APRIL molecules rank as type II membrane receptors. They exist as membrane molecules and are in soluble form. BAFF and APRIL molecules create both homotrimers and heteromers capable of stimulating B lymphocyte differentiation (Wu *et al*., 2000; Locksley *et al*., 2001). It has been proved that APRIL and BAFF molecules interfere with CSR of T-independent antibody response (Litinskiy *et al*., 2002; Martin & Vishva, 2005).

**Figure 2:**
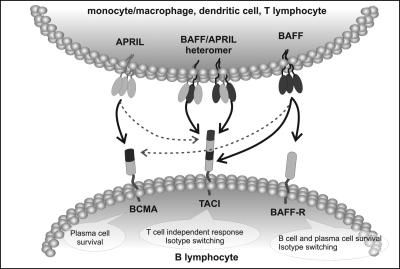
TNF receptor family members, BAFF-R, BCMA and TACI, and their ligands BAFF and APRIL check terminal differentiation of B lymphocytes and/or CSR of Ig synthesis. TNF receptors and their ligands regulate both the survival and the apoptosis during immune system development and immune responses. Dashed lines show the interactions with limited functions.

Both APRIL and BAFF molecules bind two type III membrane receptors, BCMA, TACI (Schneider *et al*., 1999). Both receptors belong to a superfamily of TNF receptors. BCMA is expressed exclusively on B lymphocytes while TACI is expressed both on B lymphocytes and activated T lymphocytes. The third receptor belonging to the TNF-R family is the BAFF-R, which is the unique receptor for BAFF (Bodmer *et al*., 2002; Losi *et al*., 2005). This membrane molecule is predominantly expressed on B lymphocytes and only on part of activated T lymphocytes (Schneider & Tschopp, 2003; Martin & Vishva, 2005). Interactions of APRIL and BAFF, expressed on monocytes and macrophages, with their receptors on B lymphocytes are evidence of the significance of natural immunity for the correct antibody response (McHeyzer-Williams, 2003; de Durana *et al*., 2006). The absence of any of the previously mentioned signals is the cause for defect of terminal differentiation of a mature B lymphocyte into plasmatic and memory cells, which are manifested by antibody immunodeficiency (Salzer & Grimbacher, 2005) (Figure 2).

Receptor TACI molecules are encoded by the TNFRSF13b gene, which is situated at the short arm of chromosome 17 (17p11.2). The described mutations affect TACI molecule in its extracellular (C104R, S144X), transmembrane (A181E) and intracellular parts (S194X, R202H, Ins204) (Castigli *et al.*, 2005b). Both homozygous and heterozygous TNFRSF13b mutations are associated with antibody deficiencies (Salzer *et al*., 2005). This suggests that TACI deficiency may manifest in autosomal dominant as well as in recessive traits in familial and in sporadic CVID. In the case of heterozygous mutations, the clear evidence that they are the cause of humoral deficiencies has not been shown. It is not possible to exclude that heterogeneous mutations may be the only modifying factor that leads to the manifestation of other defects. The TACI mutations show a range of clinical symptoms from no infection to very severe infections, suggesting that other genetic and environmental factors contribute to the variable disease spectrum. In addition, TACI deficiency may also represent a common genetic defect for CVID and selective IgA deficiency (sIgAD), which has been long proposed to be based on the clinical observations in CVID/sIgAD families (Salzer & Grimbacher, 2006).

The numbers of B cells in homozygous TACI deficiency are normal or tend to be slightly reduced. Patients with homozygous mutations in TACI showed selective impairment of APRIL and BAFF-induced B-cell proliferation and CSR. They have low numbers of CD19^+^/CD27^+^/IgM^−^ B lymphocytes and suffer from severe infections caused by encapsulated microbes. The vaccination with non-conjugated vaccines is not effective. In the case of homozygous TACI mutations, polysaccharide antigens are followed by a disturbance in the proliferation of B lymphocytes (Castigli *et al.*, 2005b).

Unlike knock-out TACI^−^/^−^ mice, in which spontaneous development of lymphomas occur, only one case of malignant lymphoma has been described in patients with TACI mutation (Seshasayee *et al*., 2003). The increased incidence of lupus-like disease seen in TACI^−^/^−^ mice was not observed in CVID patients with the same defect. In case of patients with CVID, the incidence of autoimmune manifestations in patients with and without TACI defect is the same. The incidence of TNFRSF13b mutations is estimated to be in 5–10% of patients with CVID (Salzer & Grimbacher, 2006).

## CD19 molecule deficiency

The CD19 molecule is detectable in the cytoplasm of B lymphocyte precursors, and from the stage of immature B lymphocytes it is imbedded in the cell membrane. It participates in feedback regulation of signals which come through BcR. On the other hand, it facilitates the recognition of antigen-antibody complexes that link to BcR and the CD21 molecule. Together with the CD21 molecule, it ensures another communication between natural immunity and B lymphocytes. van Zelm described the homozygous mutation in the CD19 gene in four members of a family with antibody deficiency that complies with classification criteria for CVID. The encoding gene is situated on the short arm of chromosome 16 (mapped 16p11.2) (van Zelm *et al*., 2006). The defect is autosomally recessive. Its real frequency is unknown in patients with CVID because the findings discovered up to now are rare. The numbers of peripheral B lymphocytes are normal or only slightly reduced in affected individuals. CD19 expression on B lymphocytes is missing and as a result of this, the expression of CD21 surface molecules is reduced. Calcium influx into cells is disturbed (McHeyzer-Williams, 2003). The hypersomatic mutation process proceeds in a normal way; however, CD27^+^ B lymphocytes are missing. Post-activation antibody response to rabies vaccine is insufficient. In diagnosed individuals, the defect was manifested by repeated bacterial infections. Autoimmune and lymphoproliferative symptoms were not observed (van Zelm *et al*., 2006).

## Conclusion

In 1953 Janeway described a case of a 39-year-old woman with hypogammaglobulinaemia, which is considered the first mention of CVID. More than 50 years have passed during which separate pathological units, as for example hyper IgM syndrome linked and unlinked to X-chromosome and autosomal recessive agammaglobulinaemia, have been excluded. In the last 3 years, mutations of genes encoding ICOS, TACI and CD19 have been described. These defects are found in less than 15% of the patients and for this reason the etiology of most of the patients with CVID remains unknown. The described mutations and findings of other expected gene defects will probably cause disintegration of this heterogeneous antibody deficiency syndrome.
